# Kidney injury and other complications related to colonoscopy in inpatients at a tertiary teaching hospital

**DOI:** 10.6061/clinics/2018/e456

**Published:** 2018-10-05

**Authors:** Ligia Fidelis Ivanovic, Bruno C Silva, Arnaldo Lichtenstein, Edison Ferreira de Paiva, Maria Lucia Bueno-Garcia

**Affiliations:** I Medicina Interna, Hospital das Clinicas HCFMUSP, Faculdade de Medicina, Universidade de Sao Paulo, Sao Paulo, SP, BR; II Divisao de Nefrologia, Hospital das Clinicas HCFMUSP, Faculdade de Medicina, Universidade de Sao Paulo, Sao Paulo, SP, BR

**Keywords:** Colonoscopy, Kidney Injury, Complications

## Abstract

**OBJECTIVES:**

To describe clinical complications related to colonoscopy in inpatients with multiple diseases. Among the known complications, acute kidney injury was the primary focus.

**METHODS:**

This was an observational retrospective study of 97 inpatients. Data relating to age; gender; comorbidities; current medication; blood tests (renal function, blood glucose and LDL cholesterol levels); length of hospital stay; indication, results, and complications of colonoscopies; and time to the development of kidney injury were collected between June 2011 to February 2012.

**RESULTS:**

A total of 108 colonoscopies (9 screening and 88 diagnostic) were conducted in 97 patients. Renal injury occurred in 41.2% of the patients. The univariate analysis revealed that kidney injury was related to the use of diuretics, statins, calcium channel blockers, and angiotensin converting enzyme inhibitor; however, the multivariate analysis showed that only the use of diuretics was associated with kidney injury. The occurrence of kidney injury and the time to its development were independent of the previous glomerular filtration rate as calculated with the Chronic Kidney Disease Epidemiology Collaboration (CKD-EPI) equation.

**CONCLUSIONS:**

The use of diuretics was the only independent variable associated with the development of kidney injury in inpatients with multiple comorbidities who underwent colonoscopy. The occurrence of kidney injury and the time to its development were independent of previous CKD-EPI-based assessments of renal function. These results highlight the increased risk of colonoscopy in such patients, and its indication should be balanced strictly and perhaps avoided as a screening test.

## INTRODUCTION

Colorectal cancer is the second most common cancer worldwide among women and the third among men 1. It is the third leading cause of cancer-related deaths in the United States 2. Although its incidence has declined in developed countries due to early detection of pre-malignant lesions, developing countries (e.g., countries in South America, Central America and eastern Europe) have observed an opposing trend 1,3.

Many tests are available for screening purposes; these tests include stool-based tests (e.g., faecal occult test and faecal immunochemical test), radiologic tests (e.g., computed tomographic colonography), and endoscopy-based tests (e.g., flexible sigmoidoscopy and total colonoscopy) 2. Randomized controlled trials have shown a reduction in colorectal cancer mortality when stool-based tests are used for screening 4,5. Total colonoscopy has some advantages because it permits the direct visualization of the entire colon, biopsy of suspected lesions and removal of pre-malignant lesions in both sides of colon during the same procedure 6. However, colonoscopy is an invasive method with risks related to preparation, sedation, and therapeutic procedures. Some studies have reported risks of gastrointestinal bleeding and colonic perforation related to colonoscopy 7,8. The decision-making process about which screening strategy to choose should consider invasiveness, cost, accuracy, patient age and concomitant illnesses 2,9.

Sedation during colonoscopy may lead to hypoxemia, bradycardia, hypotension, cardiac arrhythmias, or cerebrovascular events 10,11. Complications specifically related to bowel preparation include poor preparation, kidney injury and hydro-electrolyte disorders 12-14. Sodium phosphate solutions for bowel preparation have been related to increased risk of acute renal failure (acute phosphate nephropathy possibly associated with hyperphosphatemia) and delayed kidney injury 12. Some retrospective studies have found similar rates of kidney injury after colonoscopy with sodium phosphate solutions or polyethylene glycol-based solutions. Regardless of the solution used, bowel preparation may lead to acute kidney injury (AKI) and cardiovascular complications, especially among the elderly population 14,15.

Studies have compared screening strategies for colorectal cancer 4,5. In populations with several comorbidities, colonoscopy for diagnostic or therapeutic purposes can be associated with high rates of complications. However, studies focusing on clinical complications after colonoscopy among inpatients, with multiple diseases at tertiary or quaternary hospitals, are scarce in the literature 13,14. Therefore, the aim of this study was to describe clinical complication rates of inpatients subjected to colonoscopy at a tertiary teaching hospital. Among the complications assessed, AKI according to the KDIGO criteria was the primary focus 16.

## METHODS

This is an observational, retrospective cross-sectional study. Data were collected from the medical records of patients who underwent colonoscopy between June 2011 and February 2012 at an internal medicine ward at the Hospital das Clínicas da Faculdade de Medicina da Universidade de São Paulo, in São Paulo, Brazil. The study was approved by the local Ethics Committee for Research Project Analysis - Cappesq (#13208/2015). Because this was a retrospective study with no patient identifiers, informed consent from the patients was not required by the ethics committee.

Population: All patients hospitalized in the internal medicine ward for screening or diagnostic colonoscopy during the study period were included. Colonoscopy preparation using bisacodyl and mannitol was the same for all the patients.

The Hospital das Clínicas of Faculdade de Medicina da Universidade de São Paulo is the largest hospital in Latin America. It is a tertiary and quaternary public teaching hospital where undergraduate medical students and medical residents work daily. The internal medicine ward has 48 beds, and physicians in this ward treat patients with multiple comorbidities using polypharmacy to diagnose and manage unstable chronic diseases. The most frequent patient comorbidities are hypertension, diabetes mellitus, chronic pulmonary disease, cardiac failure, systemic lupus, rheumatoid arthritis, and chronic renal failure. Patients undergoing maintenance dialysis were excluded from this study.

Patients were divided in groups according to colonoscopy indication (screening and diagnostic) and renal function (creatinine clearance above or below 30 mL/min). The demographic and clinical data collected were age, comorbidities, medication use 48h before colonoscopy, blood tests (renal function, blood glucose and LDL cholesterol), indication for colonoscopy, length of hospital stay, the results and complications of colonoscopies, and time until the development of kidney injury (if it occurred).

Kidney injury was defined as a 0.3-mg/dL absolute increase in creatinine at 48 h (KDIGO 1), a 1.5- to 1.9-fold increase in basal creatinine within 7 days (KDIGO 1), a 2- to 2.9-fold increase in basal creatinine within 7 days (KDIGO 2), or a >3-fold increase in basal creatinine within 7 days (KDIGO 3) according to the KDIGO 2012 criteria 16.

### Statistical analysis

Continuous variables are expressed as either the mean ± standard deviation or the median (minimum, maximum) according to their distribution. Normality was tested with the Kolmogorov-Smirnov test and homogeneity with Levene's test. Categorical variables were expressed as proportions, and the association between kidney injury and comorbidities or medication use was tested with the chi-square test. The covariates of patients divided according to the Chronic Kidney Disease Epidemiology Collaboration (CKD-EPI) glomerular filtration rate were analysed. An unpaired t test or a Mann-Whitney test were used to compare changes in the variables between the screening and diagnostic groups as appropriate. Multivariate relationships between the diagnosis of AKI and independent variables were also examined with logistic regression. A two-tailed *p*-value <0.05 was considered statistically significant. For statistical analysis, SPSS 20 software (SPSS Inc., Chicago, IL, USA) was used.

## RESULTS

During the study period, 97 in patients underwent colonoscopy (88 diagnostic and 9 screening). Table 1 summarizes the demographics, comorbidities and regular medications of the cohort. Indications for diagnostic colonoscopies were weight loss (46.4%), change in bowel habits (34.0%), anaemia (32.0%), blood loss (13.4%), and neoplasia under investigation (4.1%).

Table 2 shows the results of the blood tests. Not all the patients had blood glucose and cholesterol levels available.

A total of 108 colonoscopies were performed in 97 patients (eleven patients underwent another colonoscopy due to inadequate preparation). Most patients (77.8%) who underwent a screening colonoscopy presented clinical complications; six presented renal injury, two had inadequate bowel preparation and one had hypoglycaemia. Among the patients in the diagnostic group, almost half presented clinical complications, most of which were due to renal injury. Inadequate bowel preparation, sepsis, hypotension, cardiovascular complications (e.g., arrhythmia and decompensation of heart failure), nausea or intestinal sub-occlusion were also present but were less frequent. In the diagnostic group, two patients could not complete their exam due to intestinal sub-occlusion (Table 3).

AKI occurred in 41.2% of the patients, most of whom were in the diagnostic group (Table 3). The mean time to kidney injury presentation after colonoscopy was less than two days with no difference between screening and diagnostic groups (1.17±1.72 *versus* 1.27±1.52 days, respectively, *p*=0.924). The incidence of colon tumours was approximately 1/3 of the studied population, but malignant cancer was rare and found solely in patients in the diagnostic group (Table 3).

There was no association between kidney injury and age (*p*=0.319); blood glucose (*p*=0.304); LDL cholesterol (*p*=0.428); comorbidities such as diabetes (*p*=0.110), cerebrovascular disease (*p*=0.471), or cardiac diseases (*p*=0.289); length of hospital stay (*p*=0.543); use of aspirin (*p*=0.390), insulin (*p*=0.108), or beta blockers (*p*=0.105); and anatomopathological findings as cancer (*p*=0.716), low grade adenoma (*p*=0.141), intermediate grade adenoma (*p*=0.928), or high grade adenoma (*p*=0.226).

The univariate analysis revealed that kidney injury was associated with the use of angiotensin converting enzyme inhibitors (ACEIs) (*p*=0.032), diuretics (*p*<0.001), calcium channel blockers (*p*=0.043) and statins (*p*=0.05) for all the patients. The associations were not tested separately for the screening and diagnostic groups due to the small size of the screening group.

The multivariate analysis only included covariates that were associated with kidney injury in the univariate analysis: the use of diuretics, statins, calcium channel blockers, and ACEIs. Age and diabetes were also included in the multivariate analysis due to their clinical relevance. Only the use of diuretics was associated with kidney injury (*p*=0.011) (age, *p*=0.955; diabetes, *p*=0.422; use of ACEIs, *p*=0.899; use of calcium channel blockers, *p*=0.621; and use of statins, *p*=0.821).

Patients were divided in four groups according to their CKD-EPI glomerular filtration rate (CKD-EPI ≥60, CKD-EPI 30-59, CKD-EPI 15-29, CKD-EPI <15 mL/min/1.73 m^2^) and analysed for continuous covariates. Only 5 patients had EPI <30 mL/min/1.73 m^2^ and were excluded from the analysis. The length of hospital stay, blood glucose, LDL and time to AKI were similar among the CKD-EPI groups (Table 4).

The indications for colonoscopy of the five patients who presented a CKD-EPI glomerular filtration rate lower than 30 mL/min/1.73 m^2^ were screening (1), change in bowel habits (2), anaemia (1), and blood loss (1). In this group, only the screening patient presented any complications (kidney injury). After we excluded these 5 patients, we compared the incidence of kidney injury and the time to AKI development between patients with CKD-EPI ≥60 and patients with CKD-EPI 30-59. The analysis showed that the occurrence of kidney injury and time to its development were independent of previous CKD-EPI glomerular filtration rate (X^2^ test *p*=0.237, Mann-Whitney test *p*=0.40 respectively).

## DISCUSSION

This study highlights three main points: the most important factor in the development of kidney injury in inpatients presenting multiple comorbidities who undergo colonoscopy is the use of diuretics; kidney injury is an early event after the exam and the occurrence of kidney injury and the time to its development were independent of the previous CKD-EPI glomerular filtration rate.

The internal medicine ward at Hospital das Clínicas is a tertiary university institution where most patients are hospitalized for diagnostic investigation or treatment of unstable chronic diseases. These individuals usually present multiple comorbidities such as hypertension, diabetes mellitus, chronic pulmonary disease, cardiac failure, systemic lupus, rheumatoid arthritis, and chronic renal failure and engage in polypharmacy (i.e., use of more than three types of medications). Therefore, the risk of complications in this population was expected to be higher than that observed in screening studies for colon cancer in asymptomatic patients in an outpatient setting 5. However, our screening group was small, which may have influenced the statistical analysis.

Some results were unexpected. Age and comorbidities such as cardiac diseases, cerebrovascular diseases, and LDL cholesterol levels were similar between the groups, but the screening group had a higher prevalence of diabetes and use of statins, ACEIs, diuretics and insulin, suggesting that these individuals have comorbidities at more advanced stages. The results of the laboratory tests indicate that creatinine and glomerular filtration rate (as measured by CKD-EPI) were similar between the groups both before and after colonoscopy.

Regarding clinical complications, AKI was the most prevalent and occurred more frequently in the screening population. The higher prevalence of diabetes in the screening group may have contributed to this difference. The higher incidence of complications in the screening group highlights their susceptibility to adverse events, and this should be noted before indicating a screening colonoscopy in older patients with multiple diseases who engage in polypharmacy. Due to their profile, this population should be hospitalized prior to undergoing screening colonoscopy, and our data suggest that doctors should carefully balance the risks and benefits of this procedure before indicating hospitalization for colonoscopy or not indicating it at all.

Bowel preparation was the same for all the patients, and none of them received oral sodium phosphate, which has been associated with AKI 12. On the other hand, a large outpatient screening study showed that AKI risk is not associated with oral sodium phosphate. The incidence of kidney injury in this setting was very low (0.2 to 0.3%). Patients were 50 to 75 years old and probably had fewer and less severe comorbidities, which permitted them to undergo colonoscopy as outpatients 17. The multivariate analysis revealed an association between kidney injury and the use of diuretics. Patients on regular diuretics use may become hypovolemic. In addition, bowel preparation for colonoscopy may lead to dehydration, a worsening hypovolemia state and renal hypoperfusion and ischaemia, which can develop into pre-renal failure due to acute tubular necrosis and AKI. There is some evidence that adequate hydration should be provided during colonoscopy, especially if the individual has reduced renal function 15. Additionally, renal function should be monitored before and after colonoscopy in patients at risk of renal dysfunction 18. Our study supports the importance of this safety practice.

The time to kidney risk or injury was similar between the screening and diagnostic groups independent of previous renal function (EPI ≥60 or EPI 30-59) and was an early event approximately 1 day after the start of bowel preparation. The literature notes that renal failure due to hypovolemia may develop within 24h or several days after colonoscopy depending on the blood volume and renal perfusion 15. Although this observation should be interpreted carefully due to the small size of the screening group, it may reflect the severity of the patients at a tertiary hospital who have an established increased high risk to AKI, which would affect the results.

Our study has some limitations. It is an observational and retrospective study that does not allow causality. We did not assess the use of other types of medications such as non-steroidal anti-inflammatory drugs or anticoagulants, which might have influenced any bleeding complications. External generalizability is limited due to the single-centre design of this study. Additionally, the entire study cohort underwent screening or diagnostic colonoscopy while hospitalized, which may reflect a population with a more severe clinical profile; thus, our results may not be suitable for an outpatient procedure. This could have introduced selection bias to our study. Finally, the small size of the screening group requires that comparisons be interpreted carefully.

However, despite the limitations, this study highlights some key points that should be considered before indicating colonoscopy to patients at tertiary or quaternary hospitals. There is an association between the use of diuretics and AKI after colonoscopy, which is probably due to exacerbated hypovolemia during bowel preparation, and anticipating renal injury in these patients may be crucial. The development of colonoscopy-related AKI occurred independent of previous renal function, which was not expected. The time to AKI development was similar in the diagnostic and screening groups and independent of previous renal function. These data support and reinforce the necessity of balancing the risk and benefits of colonoscopy in patients with multiple chronic metabolic diseases who engage polypharmacy and are older than 65 years. The decision for indicating colonoscopy as a diagnostic procedure should also be carefully considered, and the indication for screening perhaps should be avoided because the procedure may do more harm than good for these patients.

The maintenance of adequate hydration with fluid administration and the suspension of diuretics may reduce renal complications related to colonoscopy. Performing daily weight checks during bowel preparation and after colonoscopy may be an efficient and inexpensive approach to assess proper hydration and mitigate complications related to the procedure.

## AUTHOR CONTRIBUTIONS

Ivanovic LF and Bueno-Garcia ML designed the study, analyzed the data and helped drafting the manuscript. Silva BC designed the study, analyzed the data and helped drafting the manuscript. Lichtenstein A and Paiva EF designed the study and helped drafting the manuscript.

## Figures and Tables

**Table 1:** Demographics, comorbidities and medications used as stratified by the colonoscopy indication.

Covariate	All patients (N=97)	Screening group (N=9)	Diagnostic group (N=88)	*p*
Age, years (min; max)	71 (19; 87)	70 (61; 79)	71 (19; 87)	0.886
Gender, % (N)				
Male	37.4% (46)	33.3% (3)	48.9% (43)	0.374
Female	41.5% (51)	66.7% (6)	51.1% (45)	0.374
Length of hospital stay, days (min; max)	10 (3; 86)	6 (3; 14)	10 (3; 86)	0.022
Comorbidities, %				
Diabetes	45.4%	100%	39.8%	0.001
Heart disease	26.8%	22.2%	27.3%	0.745
Cerebrovascular disease	14.4%	11.1%	14.8%	0.766
Regular medications, %				
ACEI	49.5%	100%	44.3%	0.001
Diuretic	36.1%	77.8%	31.8%	0.006
Calcium channel blocker	28.9%	55.6%	26.1%	0.064
Beta blocker	30.9%	44.4%	29.5%	0.357
Aspirin	27.8%	33.3%	27.3%	0.699
Statin	36.1%	66.7%	33%	0.045
Insulin	15.1%	55.6%	11.4%	0.000

All patients: all participants in the study; Screening group: patients who underwent a screening colonoscopy; Diagnostic group: patients who underwent a diagnostic colonoscopy; N: sample size. Continuous data are expressed as the mean±SD; non-parametric data are expressed as the median (minimum, maximum).

**Table 2:** Laboratory data as stratified by colonoscopy indication.

Blood test	All patients	Screening group	Diagnostic group	*p*
Blood glucose, mg/dL (min; max)	109 (50; 437) (N=89)	203 (84; 437) (N=9)	106.5 (50; 237) (N=80)	0.001
Glycated Haemoglobin, % (min; max)	6.25 (4.7; 14.5) (N=44)	9.04 (5.3; 14.5) (N=7)	6.2 (4.7; 9.4) (N=37)	0.024
LDL cholesterol, mg/dL (min; max)	110 (45; 267) (N=60)	119 (84; 147) (N=9)	110 (45; 267) (N=51)	0.310
Creatinine pre, mg/dL	1.12±1.11	1.14±0.46	1.12±1.16	0.252
CKD-EPI pre, mL/min	71.7±28.02	60.67±20.73	72.93±28.51	0.179
Creatinine post, mg/dL	1.38±1.18	1.51±0.58	1.37±1.22	0.104
CKD-EPI post, mL/min	61.86±31.9	43.67±17.57	63.72±32.61	0.075

All patients: all participants of the study; Screening group: patients submitted to screening colonoscopy; Diagnostic group: patients submitted to diagnostic colonoscopy; N: sample size; pre, before colonoscopy; post, after colonoscopy; CKD-EPI, Chronic Kidney Disease Epidemiology Collaboration. Continuous parametric data are expressed as the mean±SD; non-parametric data are expressed as the median (minimum, maximum).

**Table 3:** Complications of colonoscopy as stratified by colonoscopy indication.

Complications	All patients (N=97)	Screening group (N=9)	Diagnostic group (N=88)	*p*
All events %, (N)	53.6% (52)	77.8% (7)	51.1% (45)	0.127
Sepsis, % (N)	2.1% (2)	0	2.3% (2)	0.648
Hypotension, % (N)	5.2% (5)	0	5.7% (5)	0.463
Cardiovascular events	2.1% (2)	0	2.3% (2)	0.648
Inadequate bowel preparation, % (N)	15.5% (15)	22.2% (2)	14.8% (13)	0.556
Suspended exam, % (N)	2.1% (2)	0	2.3% (2)	0.648
Hypoglycaemia, % (N)	1% (1)	11.1% (1)	0	0.002
Nausea/vomiting/sub-occlusion, % (N)	2.1% (2)	0	2.3% (2)	0.648
Acute kidney injury, % (N)	41.2% (40)	66.7% (6)	38.6% (34)	0.104
Time to acute kidney injury, days±SD	1.11±1.49	1.17±1.72	1.27±1.52	0.924
Cancer, % (N)	3.3% (4)	0	4.5% (4)	0.514
Low grade adenoma, % (N)	16.3% (20)	22.2% (2)	19.3% (17)	0.834
Moderate grade adenoma, % (N)	7.1% (7)	11.1% (1)	6.8% (6)	0.635
Severe grade adenoma, % (N)	2.1% (2)	11.1% (1)	11.1% (1)	0.758

All patients: all participants in the study; Screening group: patients who underwent a screening colonoscopy; Diagnostic group: patients who underwent a diagnostic colonoscopy; N: sample size.

**Table 4:** Colonoscopy-related complications according to the CKD-EPI glomerular filtration rate.

Covariate	CKD-EPI ≥60	CKD-EPI 30-59	*p*
Age, years (min; max)**	67 (19; 84)	72 (58; 85)	0.004
Length of hospital stay, days (min; max)**	11 (3; 86)	9 (3; 84)	0.22
Blood glucose, mg/dL (min; max)**	106 (50; 325)	113 (61; 252)	0.45
Glycated haemoglobin, % (min; max)**	6.2 (4.7; 9.8)	6.2 (5.1; 9.4)	0.68
LDL, mg/dL (min; max)**	119 (47; 267)	100 (45; 198)	0.17
Time to acute kidney injury, days**MedianMinimum, maximum***Mean±SD*	1-1, 51.17±1.68	1-1, 41.21±1.12	0.40

CKD-EPI: Chronic Kidney Disease Epidemiology Collaboration *Parametric data expressed as the mean±SD and tested by T Test; **non-parametric data expressed as median (minimum, maximum) and tested by Mann-Whitney test; ***Time to development of acute kidney injury, in days, considering zero as the day of the exam. Negative numbers refer to development of kidney injury before the date of the exam, during the preparation.
